# *Saccharomyces boulardii* and *Bifidobacterium* co-treatment for antibiotic associated diarrhea in pediatrics: a multicenter efficacy and safety study

**DOI:** 10.3389/fcimb.2025.1575605

**Published:** 2025-06-24

**Authors:** Qianfang Liu, Lin Liu, Jingwen Zhou, Yusong Duan, Chunlin Shi, Yan Zeng

**Affiliations:** ^1^ Department of Paediatrics, People's Hospital of Deyang City, Deyang, Sichuan, China; ^2^ Department of Paediatrics, The First People’s Hospital of Zhaotong City, Zhaotong, Yunnan, China; ^3^ Department of Paediatrics, The Third Hospital of Mianyang, Mianyang, China

**Keywords:** antibiotics induced diarrhea, *Bifidobacterium*, infants, loose stool, *Saccharomyces boulardii*, watery stool, yogurt, young children

## Abstract

**Background:**

Recent or ongoing use of antibiotics causes diarrhea. Probiotic yogurt is generally used in antibiotic-induced diarrhea as adjuvant therapy. In recent times, there have been no clear recommendations or guidelines for the course of treatment of probiotics in preventing antibiotic-induced diarrhea in infants and young children in mainland China. The objectives of the study were to evaluate the efficacy and safety of *Saccharomyces boulardii* and *Bifidobacterium* in antibiotic-induced diarrhea in infants and young children in Chinese settings.

**Methods:**

Data were collected retrospectively. Infants and children received antibiotic treatments with 50 g daily regular yogurt and 50 g daily regular yogurt for 7 days after those treatments (AY cohort, *n* = 119), or with 10 billion CFU daily *Saccharomyces boulardii* and 10 billion CFU daily *Saccharomyces boulardii* for 7 days after those treatments (AS cohort, *n* = 110), or with 10 billion CFU daily *Bifidobacterium* and 10 billion CFU daily *Bifidobacterium* for 7 days after those treatments (AB cohort, *n* = 106). Two times per day loose or watery stools were considered mild diarrhea, and three or more times per day loose or watery stools were considered severe diarrhea.

**Results:**

All infants and young children reported diarrhea after the start of any type of antibiotic treatment with probiotics. Time for the start of diarrhea after the start of antibiotic treatments with probiotics was higher in infants and children of the AS cohort than in infants and children of the AY [3 (4–3) days *versus* 1 (1–1) days, *p* < 0.001] and the AB [3 (4–3) days *versus* 2 (2–1) days, *p* < 0.001] cohorts. Twenty-four (20%), 11 (10%), and 17 (16%) infants and children reported any type of diarrhea in the AY, AS, and AB cohorts, respectively. The number of patients with reported diarrhea (mild and severe) and the number of patients who required extra anti-diarrheal treatments after antibiotic treatments in the AS cohort were fewer than those reported in the AY and the AB cohorts (*p* < 0.05 for all). Yogurt reported sneezing, runny nose, redness of the eyes, and nausea in the AY cohort (*p* < 0.05 for all comparisons). *Saccharomyces boulardii* and *Bifidobacterium* caused vomiting and nausea.

**Conclusions:**

Any type of antibiotic treatment cause diarrhea in infants and young children. Probiotics especially *Saccharomyces boulardii* co-treatments with any type of antibiotic treatment, significantly reduce incidences of diarrhea in infants and young children with manageable adverse effects.

## Introduction

Recent or ongoing use of antibiotics causes diarrhea, with loose or watery or mucous or purulent or bloody or flaky stools, and that cannot be explained by various clear reasons (Zhang et al., 2024). The incidence of diarrhea after ongoing use of antibiotics in children is 5% to 62% globally (Guo et al., 2019). The incidence of diarrhea after recent or ongoing use of antibiotics in children in China is 17% to 71% (Zheng et al., 2021). This diarrhea is because of gut dysbiosis by antibiotics (Guo et al., 2019; Lukasik et al., 2022). Intestinal flora imbalance is the main reason for antibiotic-induced diarrhea (Kim et al., 2017; Guo et al., 2019).

Supplementation of probiotics to promote the growth of dominant intestinal flora with antibiotics can prevent antibiotic-induced diarrhea in infants and young children (Guo et al., 2019). However, the effects of different probiotic preparations and treatment courses on the incidence of antibiotic-induced diarrhea in infants and young children remain debatable. In general, the European Society for Pediatric Gastroenterology, Hepatology, and Nutrition strongly recommends the *Saccharomyces boulardii* CNCM 1–745 strain to prevent antibiotic-induced diarrhea in children (Szajewska et al., 2023). In recent times, there have been no clear recommendations or guidelines for the course of treatments of probiotics in preventing antibiotic-induced diarrhea in infants and young children in mainland China (National Children’s Medical Center (Beijing) and Editorial Committee of Chinese Journal of Practical Pediatrics, 2022). Currently, among the probiotic preparations for preventing and treating diarrhea in children, *Saccharomyces boulardii* and *Bifidobacterium* tetravalent live bacteria are the most widely used (Li et al., 2021). Indian Academy of Pediatrics Consensus Guidelines recommends *Saccharomyces boulardii* as adjuvant therapy in antibiotic-induced diarrhea (Yachha et al., 2022). Moreover, probiotic yogurt is generally used in antibiotic-induced diarrhea as adjuvant therapy in adults (Velasco et al., 2019) because some of the most common probiotic strains (*Lactobacillus bulgaricus*, *Streptococcus thermophilus*, *Lactobacillus acidophilus*, and *Bifidobacterium lactis*) are found in yogurt (Al-Yami et al., 2022).

The objectives of the study were to evaluate the efficacy and safety of *Saccharomyces boulardii* and *Bifidobacterium* in antibiotic-induced diarrhea against yogurt in infants and young children in Chinese settings.

## Materials and methods

### Inclusion criteria

Infants and children aged less than 3 years with non-gastrointestinal infections who received antibiotic treatments (for any kind of infection) with probiotics as adjuvant treatments were included in the study.

### Exclusion criteria

Infants and children who did not receive probiotics were excluded from the study. Infants and children who have not complete data in hospital records of patients were excluded from the study. Infants and children who have been on antibiotic treatments in the last two months were excluded from the study. Patients with missing data were excluded.

### Design, setting, period

A retrospective study of medical record analyses of the Deyang City People’s Hospital, Deyang City, Sichuan Province, China; the First People’s Hospital of Zhaotong City, Yunnan Province, China; and the Mianyang Third People’s Hospital, China, from 1 February 2023 to 30 November 2024.

### Sample size calculations

The study was based on the assumption that after antibiotic treatments with probiotics and probiotic treatments for 7 days after those treatments, no more than 16% of infants and children had severe or mild diarrhea (primary outcome measures; effect size) (Lukasik et al., 2022). Based on this assumption and α = 0.05, β = 0.2, 80% power calculations, and a 95% confidence interval (CI), the minimum number of infants and children required in each cohort was 105 (sample size).

### Cohorts

One hundred nineteen infants and children received antibiotic treatments with regular yogurt and regular yogurt for 7 days after those treatments (50 g daily; AY cohort) (Velasco et al., 2019). One hundred ten infants and children received antibiotic treatments with *Saccharomyces boulardii* and *Saccharomyces boulardii* for 7 days after those treatments (10 billion Colony Forming Units (CFU) daily; in powder form; AS cohort) (Lukasik et al., 2022). One hundred six infants and children received antibiotic treatments with *Bifidobacterium* and *Bifidobacterium* for 7 days after those treatments (10 billion CFU daily in powder form; AB cohort) (Zhang et al., 2024). The decision of yogurt, *Saccharomyces boulardii*, or *Bifidobacterium* co-treatments is the decision of the pediatrician. Infants and children have not consumed other probiotic preparations than recommended or foods containing probiotics during antibiotic treatments and 7 days of prescribed probiotic treatments after those treatments. Yogurt was given along with food to children. Parents were informed and involved in administering the yogurt. The use of probiotics or yogurt alongside antibiotics was a standard practice in the study setting. There were ~3h differences between antibiotic and probiotic administration.

## Outcome measures

### Demographical and clinical characteristics

Demographical and clinical characteristics before the start of antibiotic treatments were collected from patients’ records of institutes and analyzed.

### Diarrhea (primary outcome measures)

Two or more loose or watery stools per day caused by unexplained etiology are considered diarrhea (Lukasik et al., 2022). Two times per day loose or watery stools were considered mild diarrhea, and three or more times per day loose or watery stools were considered severe diarrhea (Lukasik et al., 2022).

### Adverse effects (secondary outcome measures)

Any adverse effects during treatments of antibiotic therapies and 7 days after the use of probiotics were collected from patients’ records of institutes and analyzed.

### Clinical benefits of probiotics

The clinical benefits of probiotic co-treatment for antibiotic-associated bacteria in infants and children who underwent antibiotic treatments were evaluated as a function of the beneficial scores. Beneficial scores for probiotic co-treatment for antibiotic-associated bacteria were calculated from the risk of under-treatment (for antibiotics-associated bacteria), as expressed in **Equation 1**. The risk of undertreatment was defined by a calculation that involved the % prevalence of severe or mild diarrhea after antibiotic treatments with probiotics and 7 days of probiotic treatment after those treatments (**Equation 2**). The % prevalence of severe or mild diarrhea after antibiotic treatments with probiotics and 7 days of probiotic treatment after those treatments ranged from 0% to 100%. The beneficial score of co-treatment of the probiotics for antibiotic-associated bacteria is the area above the curve of the adopted co-treatment for antibiotic-associated bacteria, and the working area is the area under the curve of the adopted co-treatments for antibiotic-associated bacteria. For all adopted co-treatments, the 16% prevalence of severe or mild diarrhea after antibiotic treatments with probiotics and 7 days of probiotic treatments after those treatments were used as the reference standard (Jian et al., 2019).


(1) $$\begin{array}{l} \text{Beneficial}\;\text{score}=\frac{\text{Numbers}\;\text{of}\;\text{infants}\;\text{and}\;\text{children}\;\text{without}\;\text{diarrhea}}{\text{The}\;\text{total}\;\text{number}\;\text{of}\;\text{infants}\;\text{and}\;\mathrm{children}\;\text{in}\;\text{that}\;\text{cohort}}-\\ \left(\frac{\text{Numbers}\;\text{of}\;\text{children}\;\mathrm{and}\;\text{infants}\;\text{with}\;\mathrm{diarrhea}}{\text{The}\;\text{total}\;\text{number}\;\mathrm{of}\;\text{infants}\;\text{and}\;\text{children}\;\mathrm{in}\;\text{that}\;\text{cohort}}\times \mathrm{Risk}\;\mathrm{of}\;\text{undertreatment}\right) \end{array}$$

(2) $$\text{Risk}\;\text{of}\;\text{undertreatment =}\frac{\text{The}\;\%\;\text{of}\;\text{infants}\;\text{and}\;\text{children}\;\text{with}\;\text{diarrhea}}{100\;-\text{the}\;\%\;\text{of}\;\text{infants}\;\text{and}\;\text{children}\;\mathrm{with}\;\mathrm{diarrhea}}$$

### Statistical analyses

InStat 3.01 (GraphPad Software, San Diego, CA, USA) was used for statistical analyses. Categorial normally distributed continuous, and non-normally distributed continuous variables are depicted as frequencies with percentages in parentheses, mean ± standard deviation (SD), and median with Q3–Q1 in parentheses, respectively. Fisher’s exact test or chi-square test (χ^2^-test with or without Yate’s corrections or independence) was preferred for categorical variables. Kolmogorov and Smirnov methods were used to check the normality of continuous variables. The Bartlett test was used to check the homogeneity of SDs of normally distributed continuous data. Kruskal–Wallis’ test [nonparametric analysis of variance (ANOVA)] was used for statistical analysis of non-normally distributed continuous variables. Post-hoc tests were calculated if the *p*-value was less than 0.05. Dunn’s multiple comparisons test was performed for post-hoc analysis of non-normally distributed continuous variables. All results were considered significant if the *p*-value was less than 0.05 at 95% CI (either one-tail or two-tails).

## Results

### Study populations

**Figure 1:**
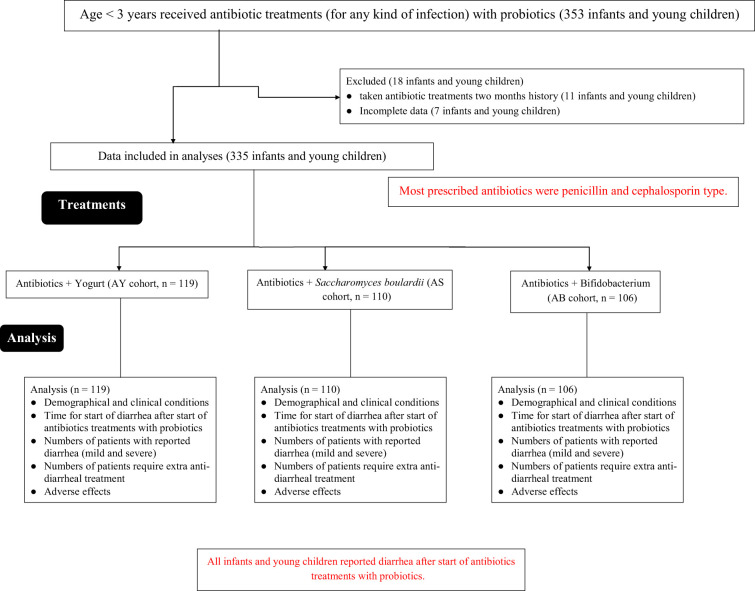
The flow diagram of the retrospective analyses. Red color indicates worse parameter.

From 1 February 2023 to 30 November 2024, a total of 353 infants and children aged less than 3 years with non-gastrointestinal infections received antibiotic treatments (for any kind of infection) with probiotics as adjuvant treatments at the Deyang City People’s Hospital, Deyang City, Sichuan Province, China; the First People’s Hospital of Zhaotong City, Yunnan Province, China; and the Mianyang Third People’s Hospital, China. Among them, 11 infants and children had taken antibiotic treatments with or without probiotics in the last two months. Data on seven infants and children were not completely available in the parent institute and the referring institutes. Therefore, data from these patients (18 infants and children) were excluded from the study. Different variables of 335 infants and young children regarding numbers of infants and children with diarrhea after antibiotics treatments with probiotics and 7 days of probiotic treatments after those treatments, duration of diarrhea since the start of antibiotics treatments, use of any other anti-diarrheal treatment after treatments, and adverse effects during and after treatments were extracted from hospital records and analyzed. The flow diagram of the retrospective analyses is presented in **Figure 1**.

### Demographical and clinical characteristics

Male-to-female ratio was almost 1:1 among all cohorts. Infants and children were 3 months to 3 years old. Infants and children had mostly fever, colic pain, tonsillitis, upper respiratory tract infection, lower respiratory tract infection, and/or injuries leading to antibiotic use. Most infants and children were of Han Chinese ethnicity, and the reported maximum type of antibiotic treatment was amoxicillin plus clavulanic acid followed by cefixime (the dose of antibiotics was according to body mass index; most prescribed antibiotics were penicillin and cephalosporin type). The most prescribed drug, other than antibiotic treatments with antibiotics was paracetamol. Sex, age, ethnicity, antibiotic treatments, and the type of other medications prescribed with antibiotics were comparable for patients among cohorts (*p* > 0.05 for all comparisons). The details of the demographical and clinical characteristics of the enrolled infants and children are presented in **Table 1**.

**Table 1:** Demographical and clinical characteristics of the enrolled infants and children in outpatient clinics of the pediatrics departments.

Parameters		Cohorts			Comparisons between cohorts		
		AY	AS	AB			
Co-treatment for antibiotics induced diarrhea		Yogurt	*Saccharomyces boulardii*	*Bifidobacterium*			
Numbers of infants and children		119	110	106	*p*-value	df	Test value
Sex	Male	50(42)	50(45)	46(43)	0.8707 (χ^2^ test of independence)	2	0.2769
Female	69(58)	60(55)	60(57)				
Age (months)		24(29–14)	21(29–15)	20(28–13)	0.6094 (Kruskal–Wallis’ test)	N/A	0.9906
Ethnicity							
Han Chinese		108(91)	99(90)	96(89)	0.9993 (χ^2^ test of independence)	4	0.07425
Mongolian		10(8)	10(9)	10(10)			
Tibetan		1(1)	1(1)	1(1)			
Antibiotics treatments							
Amoxycillin + clavulanic acid		61(51)	58(53)	46(43)	0.5965 (χ^2^ test of independence)	6	4.597
Cefixime		25(21)	30(27)	30(28)			
Cefpodoximes		15(13)	15(14)	20(19)			
Cefuroxime		7(6)	7(6)	10(9)			
Other medications prescribed with antibiotics							
Paracetamol		80(67)	78(71)	85(80)	0.9998 (χ^2^ test of independence)	8	0.5518
Mefenamic acid		30(25)	30(27)	31(29)			
Simethicone		25(21)	25(23)	28(26)			
Chlorpheniramine		11(9)	11(10)	9(8)			
B-complex drops		7(6)	7(6)	8(8)			

Categorial and non-normally distributed continuous variables are depicted as frequencies with percentages in parenthesis and median with Q3–Q1 in parenthesis, respectively.

df, degree of freedom; N/A, not applicable; CI, confidence interval; χ^2^ test: chi-square test.

Test value (χ^2^ value for χ^2^ tests; Kruskal–Wallis’ statistics for Kruskal–Wallis’ test).

All results were considered significant if the *p*-value was less than 0.05 at 95% CI.

### Outcome measures

**Figure 2:**
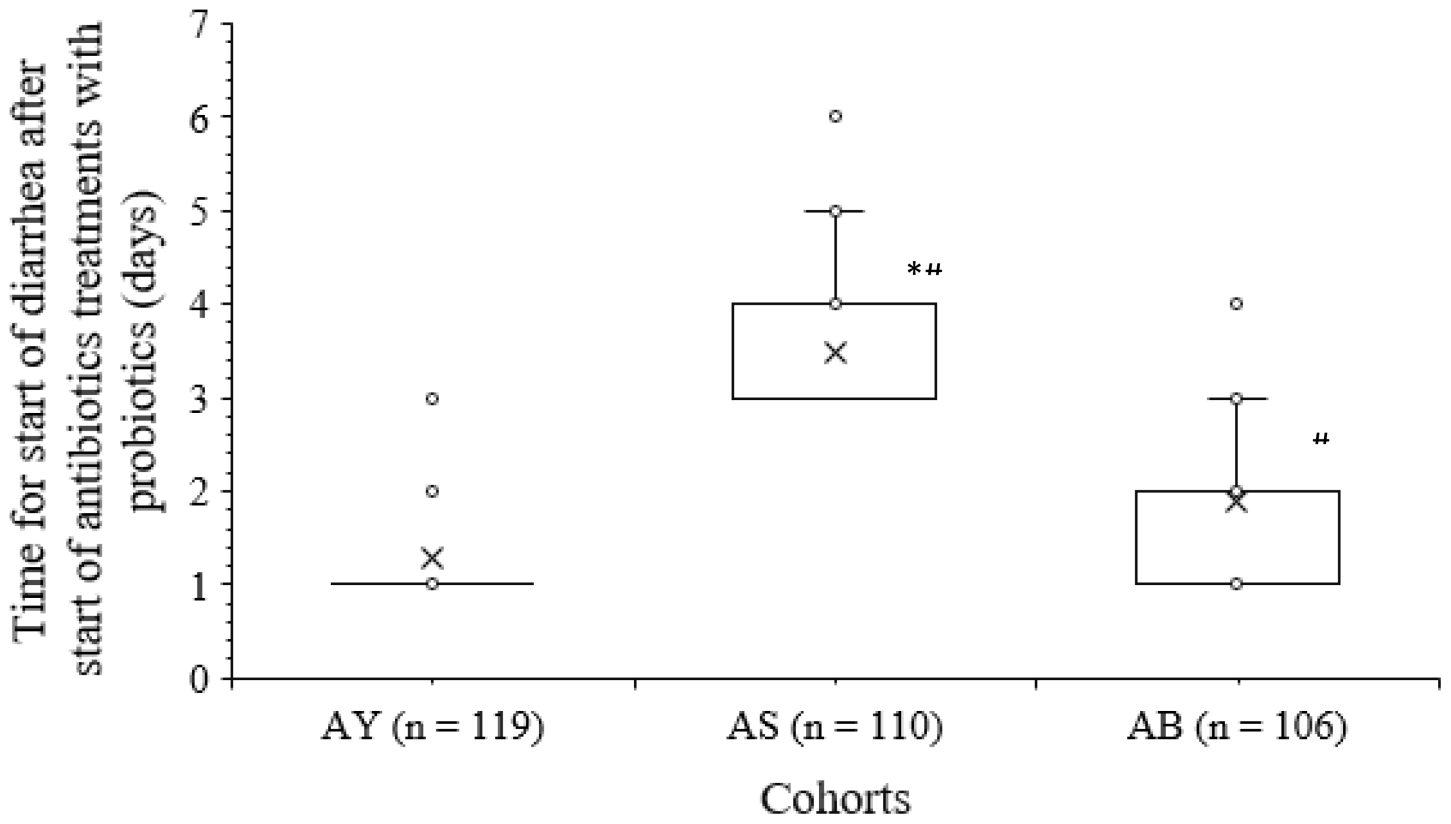
The details of the time for the start of diarrhea after the start of antibiotics treatments with probiotics in infants and children of the different cohorts. The upper line of the box indicates the third quartile value, the lower line of the box indicates the first quartile value, and “x” indicates the median value. *p < 0.001 compared to that of AB cohort; ^#^ *p* < 0.001 compared to that of AY cohort.

All infants and young children reported diarrhea after the start of antibiotic treatments with probiotics. Time for start of diarrhea after start of antibiotic treatments with probiotics was higher in infants and young children of the AS cohort than those of infants and children of the AY cohort (3 (4–3) days *versus* 1 (1–1) days, *p* < 0.001, Kruskal–Wallis’ test/Dunne’s test, Kruskal–Wallis’ statistics: 209) and the AB (3 (4–3) days *versus* 2 (2–1) days, *p* < 0.001, Kruskal–Wallis’ test/Dunne’s test, Kruskal–Wallis’ statistics: 209) cohorts. Time for the start of diarrhea after the start of antibiotic treatments with probiotics was higher in infants and children of the AB cohort than in infants and children of the AY cohort (*p* < 0.001, Kruskal–Wallis’ test/Dunne’s test, Kruskal–Wallis’ statistics: 209). The details of the time for the start of diarrhea after the start of antibiotic treatments with probiotics in infants and children of the different cohorts are presented in **Figure 2**.

Fifteen (13%), 3 (3%), and 10 (9%) patients reported mild diarrhea after antibiotic treatments with probiotics and probiotic treatments for 7 days after those treatments in the AY, AS, and AB cohorts, respectively. Nine (8%), 1 (1%), and 7 (7%) patients reported severe diarrhea after antibiotic treatments with probiotics and probiotic use for 7 days after those treatments in the AY, AS, and AB cohorts, respectively. Twenty-four (20%), 11 (10%), and 17 (16%) patients reported any type of diarrhea after antibiotic treatments with probiotics and probiotic treatments for 7 days after those treatments in the AY, AS, and AB cohorts, respectively. The number of patients with reported diarrhea (mild and severe) and the number of patients who require extra anti-diarrheal treatments after antibiotic treatment with Saccharomyces boulardii and Saccharomyces boulardii for 7 days after those treatments in the AS cohort were fewer than those reported with diarrhea (mild and severe) and numbers of patients requires extra anti-diarrheal treatments after antibiotics treatment with regular yogurt and regular yogurt for 7 days after those treatments in the AY cohort and those reported with diarrhea (mild and severe) and numbers of patients require extra anti-diarrheal treatments after antibiotics treatments with Bifidobacterium and Bifidobacterium for 7 days after those treatments in the AB cohort. The number of patients who reported diarrhea (mild and severe) and the number of patients who required extra anti-diarrheal treatments after antibiotic treatments with probiotics and probiotic use for 7 days after those treatments were statistically the same between the AY and the AB cohort. None of the young children and infants reported diarrhea after the use of anti-diarrheal treatment(s) (if required). The details of outcome measures after antibiotic treatments with probiotics and use of probiotic treatment for 7 days after those treatments of the enrolled infants and children are presented in **Table 2**.

**Table 2:** Outcome measures after antibiotics treatments with probiotics and use of probiotics course treatments for 7 days after those treatments of the enrolled infants and children.

Parameters	Cohorts											
	AY	AS				AB						
Co-treatment for antibiotics induced diarrhea	Yogurt	*Saccharomyces boulardii*	Comparisons concerning the AY cohort			*Bifidobacterium*	Comparisons concerning the AY cohort			Comparisons between AS and AB cohorts		
Numbers of infants and children	119	110	*p*-value	Relative risk	95% CI	106	*p*-value	Relative risk	95% CI	*p*-value	Relative risk	95% CI
Number of patients reported mild diarrhea after treatment	15(13)	3(3)	0.0061	1.691	1.320 to 2.166	10(9)	0.5267	1.154	0.8158 to 1.632	0.0467	0.4378	0.1609 to 1.192
Number of patients reported severe diarrhea after treatment	9(8)	1(1)	0.02	1.784	1.397 to 2.278	7(7)	0.7991	1.089	0.6936 to 1.711	0.0295	0.2332	0.03711 to 1.465
Number of patients require extra anti-diarrheal treatment	12(10)	0(0)	0.0004	2.028	1.772 to 2.321	5(5)	0.1397	1.372	0.9824 to 1.917	0.0271	0	-Infinity to Infinity

Mild diarrhea: Two times per day loose or watery stools.

Severe diarrhea: Three or more times per day loose or watery stools.

Variables are depicted as frequencies with percentages in parentheses.

CI, Confidence interval (using the approximation of Katz.).

Fisher’s exact test was used for statistical analysis.

All results were considered significant if the *p*-value was less than 0.05 at 95% CI.

### Adverse effects

During antibiotic treatments with probiotics, probiotic treatments for 7 days after those treatments, and treatments for diarrhea (if required) of the enrolled infants and children, they generally reported sneezing, runny nose, redness of eyes, vomiting, and nausea. Yogurt reported sneezing, runny nose, redness of eyes, and nausea among infants and children of the AY cohort. *Saccharomyces boulardii* and *Bifidobacterium* caused vomiting and nausea among infants and children. The details of adverse effects during antibiotics treatment with probiotics, probiotic treatments for 7 days after those treatments, and treatments for diarrhea (if required) of the enrolled infants and children are reported in **Table 3**. The results of the assumptions test adopted in the study are presented in **Table 4**.

**Table 3:** Adverse effects during antibiotics treatment with probiotics, probiotics co-treatments for 7 days after those treatments, and treatments for diarrhea (if required) of the enrolled infants and children.

Events	Cohorts											
	AY	AS				AB				Comparisons between AS and AB cohorts		
Co-treatment for antibiotics induced diarrhea	Yogurt	*Saccharomyces boulardii*	Comparisons concerning the AY cohort			*Bifidobacterium*	Comparisons concerning the AY cohort					
Numbers of infants and children	119	110	*p*-value	Relative risk	95% CI	106	*p*-value	Relative risk	95% CI	*p*-value	Relative risk	95% CI
Sneezing	65(55)	0(0)	< 0.0001	3.037	2.441 to 3.779	0(0)	< 0.0001	2.963	2.385 to 3.682	N/A	N/A	N/A
Running nose	45(38)	0(0)	< 0.0001	2.486	2.085 to 2.966	0(0)	< 0.0001	2.432	2.042 to 2.897	N/A	N/A	N/A
Redness of eyes	12(10)	0(0)	0.004	2.028	1.772 to 2.321	0(0)	0.004	1.991	1.742 to 2.275	N/A	N/A	N/A
Skin rashes	7(6)	2(2)	0.1741	1.528	1.053 to 2.218	3(3)	0.3411	1.344	0.8780 to 2.057	0.6788	0.7815	0.2649 to 2.305
Vomiting	12(10)	7(6)	0.3477	1.234	0.8541 to 1.783	5(5)	0.2055	1.366	0.9784 to 1.907	0.7684	1.155	0.7027 to 1.900
Nausea	27(23)	18(16)	0.2478	1.2	0.9079 to 1.586	19(18)	0.4109	1.142	0.8620 to 1.513	0.8751	0.9465	0.6601 to 1.357
Gag reflex	3(3)	8(7)	0.1243	0.5125	0.1937 to 1.356	5(5)	0.4801	0.7015	0.2842 to 1.731	0.5699	1.225	0.7800 to 1.923
Abdominal pain	7(6)	5(5)	0.7705	1.13	0.6887 to 1.855	4(4)	0.5464	1.216	0.7639 to 1.935	0.9999	1.095	0.6012 to 1.995
Trace of blood in the stool	1(1)	1(1)	0.9999	0.9619	0.2391 to 3.869	1(1)	0.9999	0.9449	0.2349 to 3.800	0.9999	0.9817	0.2439 to 3.951

Variables are depicted as frequencies with percentages in parentheses.

CI, Confidence interval (using the approximation of Katz.), N/A: not applicable.

Fisher’s exact test was used for statistical analysis.

All results were considered significant if the *p*-value was less than 0.05 at 95% CI.

**Table 4:** The results of assumption tests adopted in the study.

Variables	Adopted test with results and desecrations of authors regarding statistical test adopted
Categorial variables	
2 × 2 Table	Fisher’s exact test or Chi-square test (for sample size > 5 and total sample population ≥ 40) with Yate’s corrections if any individual sample was less than 10.
Large Tables	Chi-square test with independence (when all expected values are greater than 1)
Continuous variables	
Age (months)	Two columns were failed in normality tests (Kolmogorov and Smirnov). *P*-values were 0.0419, > 0.1, and 0.0779. Therefore, Kruskal–Wallis’ test (nonparametric analysis of variance (ANOVA)) was performed.
Time for the start of diarrhea after start of antibiotics treatment (days)	All columns were failed in normality tests (Kolmogorov and Smirnov). *P*-values were < 0.0001 for all. Therefore, Kruskal–Wallis’ test

### Clinical benefits of probiotics

**Figure 3:**
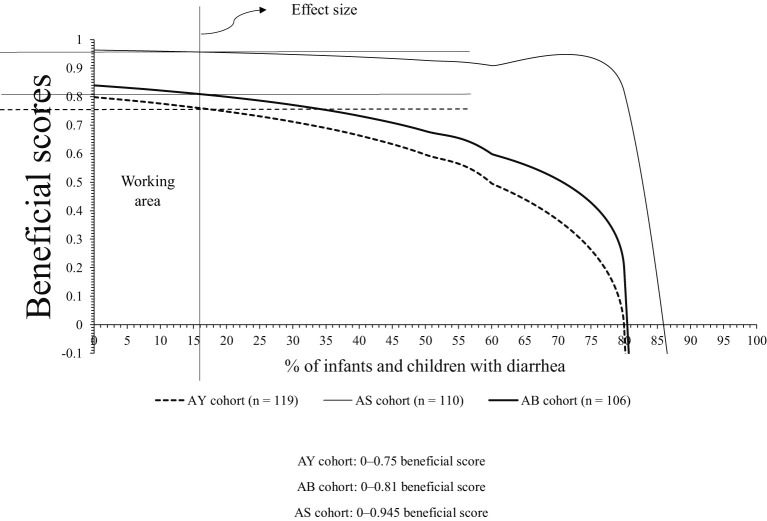
Graphical presentation of the clinical benefits of probiotics.

Clinical benefits for infants and children of the AS, AB, and AY cohorts were 0–0.95 beneficial score, 0–0.81 beneficial score, and 0–0.75 beneficial score, respectively. Above 0.95 beneficial score, 0.81 beneficial score, and 0.75 beneficial score, infants and children of the AS, AB, and AY cohorts had a risk of under co-treatment for antibiotic-associated bacteria or risk of development of diarrhea. A graphical presentation of the clinical benefits of probiotics is presented in **Figure 3**. The details of the clinical benefits of probiotics are presented in **Table 5**.

**Table 5:** The details of the clinical benefits of probiotics in the enrolled infants and children.

Parameters		Cohorts		
		AY	AS	AB
Co-treatment for antibiotics induced diarrhea		Yogurt	*Saccharomyces boulardii*	*Bifidobacterium*
Numbers of infants and children		119	110	106
Numbers of infants and children without diarrhea		95	106	89
Numbers of infants and children with diarrhea		24	4	17
% of infants and children with diarrhea	Risk of undertreatment	Beneficial score		
0	0	0.8	0.96	0.84
1	0.01	0.78	0.96	0.84
5	0.05	0.79	0.96	0.83
10	0.11	0.78	0.96	0.82
20	0.25	0.75	0.95	0.8
30	0.43	0.71	0.95	0.77
40	0.67	0.66	0.94	0.73
50	1	0.6	0.93	0.68
60	1.5	0.5	0.91	0.6
80	4	−0.01	0.82	0.2
99	99	−19.17	−2.64	−15.04
Clinical benefits (beneficial score)		0–0.75	0–0.95	0–0.81
Risk of under co-treatment for antibiotics-associated bacteria or risk of development of antibiotics induced diarrhea (beneficial score)		> 0.75	> 0.95	> 0.81

Effect size: no more than 16% of infants and children had severe or mild diarrhea after antibiotics treatments with probiotics and 7 days probiotics treatment after those treatments.

## Discussions

All infants and young children reported diarrhea after the start of antibiotic treatments with probiotics. The intestinal flora is composed of trillions of microorganisms, including bacteria, fungi, single-cell organisms, and viruses (Senchukova, 2023). Most prescribed antibiotics were penicillin or cephalosporin types in the enrolled infants and young children. Antibiotic treatments, especially from the penicillin and cephalosporin groups, can disrupt the colonization resistance of the gastrointestinal flora in infants and young children, resulting in diarrhea (Guo et al., 2019; de Nies et al., 2023), because, unlike adults, infants and young children have a simple intestinal flora structure, incomplete intestinal barrier development, low immune function, and a simple diet (Zhang et al., 2022). Antibiotic treatments cause diarrhea in infants and young children.

The time for the start of diarrhea after the start of antibiotic treatments with probiotics was higher in infants and young children of the AS cohort than in infants and children of the AY and AB cohorts. In addition, the time for the start of diarrhea after the start of antibiotic treatments with probiotics was higher in infants and children of the AB cohort than in infants and children of the AY cohort. Moreover, infants and young children of the AY cohort required a minimum of one day for incidences of diarrhea. The disruption of the gastrointestinal flora in infants and young children is managed by probiotics by directly or indirectly regulating the structure of the microbiota, interacting with intestinal epithelial cells, macrophages, and lymphocytes to repair and strengthen the intestinal mucosal barrier functions (Mekonnen et al., 2020). Probiotics increase the content of intestinal endocrine immunoglobulins and exert an intestinal immune regulatory effect (Li et al., 2023; Zhang et al., 2024). The use of antibiotics for the treatment of any disease and the addition of probiotics can effectively prevent antimicrobial-associated diarrhea, reduce the average duration of diarrhea, and reduce the average number of diarrhea episodes per day in infants and young children.

The number of patients with reported diarrhea (mild and severe) and the number of patients who required extra anti-diarrheal treatment after antibiotic treatments in the AS cohort were fewer than those reported in the AY and AB cohorts. Even clinical benefits for infants and young children of the AS cohort were higher than those of the AY and AB cohorts. The results of incidences of diarrhea are consistent with those of trials (Lukasik et al., 2022; Zhang et al., 2024). Unlike yogurt and *Bifidobacterium*, *Saccharomyces boulardii* deactivates multiple signaling pathways (NF-κβ, ERK1/2, and JNK) and decreases inflammation of intestinal inflammation (Justino et al., 2020). In addition, the poor efficacy of *bifidobacteria* is related to the number of bacteria administered or inactivated by antibiotics, as *Bifidobacterium* is sensitive to these antibiotics, while the fungus *Saccharomyces boulardii* is not sensitive. *Saccharomyces boulardii* co-treatments with antibiotic treatments significantly reduce incidences of diarrhea in infants and young children more than yogurt and *Bifidobacterium*.

None of the infants and young children had serious adverse effects. The results of the adverse effects of the current study are consistent with those of trials (Lukasik et al., 2022; Zhang et al., 2024). Probiotic co-treatments are safe for the prevention and treatment of diarrhea in infants and young children.

A minimum of 50 g of daily yogurt and 10 billion CFU of daily probiotics were recommended doses by a pediatrician. Generally, probiotics have a moderate protective effect on antibiotic-associated diarrhea. Therefore, pediatrics recommended 10 billion CFU doses because 5 billion CFUs per day or higher doses of probiotics were reported effective in antibiotic-associated diarrhea in pediatrics (Guo et al., 2019). Regular yogurt contains about 10 billion CFUs of probiotic bacteria per 50 g (Nyanzi et al., 2021).

*Saccharomyces boulardii* is a well-researched probiotic yeast, while *Bifidobacterium* tetravalent typically includes a combination of *Bifidobacterium bifidum*, *Bifidobacterium longum*, *Bifidobacterium breve*, and *Bifidobacterium infantis*. On the other hand, yogurt probiotics generally consist of live beneficial bacteria, primarily *Lactobacillus* and *Bifidobacterium* species. Given that yogurt already contains certain *Bifidobacterium* strains, however, the current study has tested the efficacy and safety of *Bifidobacterium* against yogurt, as they share overlapping bacterial components. The possible justification for the same is that the content of yogurt is varied (Fox et al., 2015), and there is no fixed amount for any strain (Nyanzi et al., 2021). In addition, yogurt is traditionally used for antibiotic-induced diarrhea (Velasco et al., 2019).

In the limitations of the study, for example, the etiology of diarrhea was not pathologically (Lukasik et al., 2022) evaluated. The cost burden due to co-treatments of probiotics with antibiotics (Yuncui et al., 2020) is not evaluated. The results of the number of patients who reported diarrhea (mild and severe), the number of patients who required extra anti-diarrheal treatments after antibiotic treatments with probiotics and probiotic treatments for 7 days after those treatments, and unwanted adverse effects between the infants and young children of the AB cohort were not reported as significant compared to those results of the AY cohort. The possible justifications for the same are the small sample size, the older average age of the children, and the high proportion of antibiotics. In addition, yogurt provides mild to moderate efficacy against antibiotic-associated diarrhea in children (Fox et al., 2015). The pH of the stomach of infants is different than that of an adult. It is also different among infants based on food habits. The current study has not correlated their results with the stomach pH of the subjects. The pH of stool also should be recorded to reveal the impact of yogurt on the gastrointestinal tract. This will reflect the gut environment before and after feeding. However, the current study has not measured the pH of stool. In addition, this is not trial and a control arm is not possible retrospective study (the same with untreated (no antibiotic) subject were not possible to be carried out). The retrospective design cannot indicate the causal relationship between parameters. There are many confounding factors that need clarification; it is difficult to reach with conclusion of this manuscript yet. Retrospective design precludes causal conclusions. Self-reported outcomes are unreliable in infants. The details of comparative studies on co-treatments of probiotics with antibiotics to overcome associated diarrhea in different settings are presented in **Table 6**.

**Table 6:** Comparative studies on co-treatments of probiotics with antibiotics to overcome associated diarrhea in different settings.

Study	Published year	Children ethnicity	Sample size (N; children)	Age	Types of probiotics	Inoculation number of probiotics	Follow-up	Main conclusions
In a prospective randomized controlled study, Zhang et al. (2024)	2024	Chinese	182	< 3 years	*Saccharomyces boulardii* and *Bifidobacterium*	10 billion Colony Forming Units	21 days	Both are effective
Randomized clinical trial, Lukasik et al. (2022)	2022	The Netherlands	350	3 months to 18 years	*Bifidobacterium bifidum* W23, *Bifidobacterium lactis* W51, *Lactobacillus acidophilus* W37, *L acidophilus* W55, *Lacticaseibacillus paracasei* W20, *Lactiplantibacillus plantarum* W62, *Lacticaseibacillus rhamnosus* W71, and *Ligilactobacillus salivarius* W24	10 billion Colony Forming Units	During the period of antibiotic treatment and for 7 days afterward	A multispecies probiotic is effective
Randomized clinical trial, Velasco et al. (2019)	2019	Spain	314	75.08 ± 14.6 years	Probiotic yogurt	200 ml	4 weeks	Probiotic yogurt is effective
Randomized clinical trial, Fox et al. (2015)	2015	Australia	70(36:34)	1–12 years	Probiotic yogurt	200 g/day	Antibiotics treatment plus 1 week	Probiotic yogurt is effective

## Conclusions

Any type of antibiotic treatment causes diarrhea in infants and young children. The addition of probiotics can effectively prevent any type of antimicrobial-associated diarrhea for the treatment of any disease, reduce the average duration of diarrhea, and reduce the average number of diarrhea episodes per day in infants and young children. *Saccharomyces boulardii* co-treatments with any type of antibiotic treatment significantly reduce incidences of diarrhea in infants and young children more than yogurt and *Bifidobacterium*. Probiotic co-treatments are safe for the prevention and treatment of diarrhea in infants and young children.

## Data Availability

The original contributions presented in the study are included in the article/**Supplementary Material**. Further inquiries can be directed to the corresponding author.
